# Treatments for hearing loss in osteogenesis imperfecta: a systematic review and meta-analysis on their efficacy

**DOI:** 10.1038/s41598-022-20169-9

**Published:** 2022-10-12

**Authors:** Maialen Ugarteburu, Luis Cardoso, Claus-Peter Richter, Alessandra Carriero

**Affiliations:** 1 Department of Biomedical Engineering, The City College of New York, New York, NY USA; 2 Department of Otolaryngology, Northwestern University Feinberg School of Medicine, Chicago, IL USA; 3 Department of Biomedical Engineering, Northwestern University, Evanston, IL USA; 4 Department of Communication Sciences and Disorders, Northwestern University, Evanston, IL USA; 5 The Hugh Knowles Center, Northwestern University, Evanston, IL USA

**Keywords:** Osteogenesis imperfecta, Outcomes research, Auditory system

## Abstract

About 70% of people with osteogenesis imperfecta (OI) experience hearing loss. There is no cure for OI, and therapies to ameliorate hearing loss rely on conventional treatments for auditory impairments in the general population. The success rate of these treatments in the OI population with poor collagenous tissues is still unclear. Here, we conduct a systematic review and meta-analysis on the efficacy of treatments addressing hearing loss in OI. This study conforms to the reporting standards of the Preferred Reporting Items for Systematic Reviews and Meta-analyses (PRISMA). Data sources include published articles in Medline via PubMed, Web of Science, Scopus, and Embase, from their inception to November 2020. Studies included individuals with OI undergoing a hearing loss treatment, having pre- and postoperative objective assessment of hearing function at a specified follow-up length. Our search identified 1144 articles, of which 67 were reviewed at full-text screening. A random-effects meta-analysis was conducted on the selected articles (n = 12) of people with OI that underwent stapes surgery. Success was assessed as the proportion of ears with a postoperative Air–Bone Gap (ABG) ≤ 10 dB. A systematic review was conducted on the remaining articles (n = 13) reporting on other treatments. No meta-analysis was conducted on the latter due to the low number of articles on the topic and the nature of single case studies. The meta-analysis shows that stapes surgeries have a low success rate of 59.08 (95% CI 45.87 to 71.66) in the OI population. The systematic review revealed that cochlear implants, bone-anchored hearing aids, and other implantable hearing aids proved to be feasible, although challenging, in the OI population, with only 2 unsuccessful cases among the 16 reviewed single cases. This analysis of published data on OI shows poor clinical outcomes for the procedures addressing hearing loss. Further studies on hearing loss treatments for OI people are needed. Notably, the mechanisms of hearing loss in OI need to be determined to develop successful and possibly non-invasive treatment strategies.

## Introduction

Hearing loss is common in people with osteogenesis imperfecta (OI or *brittle bone disease*), a genetic disease of the connective tissues caused mainly by mutations in collagen type I^1^. Clinical features are brittle bones with spontaneous fractures, skeletal deformities, joint laxity, blue sclerae, dentinogenesis imperfecta, cardiovascular and respiratory problems, and hearing loss^2^. Progressive hearing loss affects about 70% of people with OI^3–9^, with a prevalence of cases happening already in childhood^5,9–18^. The incidence of hearing loss in OI is notably higher than in the general population, which according to the World Health Organization, is just over 5% of the world’s population^19^. Interestingly, bone fracture rate decreases after puberty in OI, but hearing loss worsens with age^6,9,15^.

Hearing loss in OI is often bilateral^20,21^ and can be conductive, sensorineural, or mixed, with 50% of the cases involving the inner ear^3–8^. To date, the relationships between the type of OI, or genetic mutation, and onset, type and severity of hearing loss are still unclear. Among the four types of classical OI described by Sillence et al.^22^, type I (mild), type II (lethal), type III (severe), and type IV (moderate-to-severe), the author reported hearing loss as a common symptom of OI type I and less frequent in OI type IV^23^. Different observations come from Hartikka et al.^7^ and Swinnen et al.^8^, who found no correlation between the mutated gene and OI type and the severity of hearing loss. Instead, da Costa Otavio et al.^12^ reported normal hearing thresholds in people with OI type I, conductive hearing loss for OI type III, and sensorineural hearing loss for OI type IV. In a multicenter study, Machol et al.^24^ found an increased prevalence for hearing loss with age in OI type I, while people with OI type III and IV are at higher risk of developing hearing loss already in the first decade of life. The authors also reported a prevalence of sensorineural hearing loss in female individuals with OI^24^, although hearing loss in OI was previously reported as gender independent^20,21^. Disparities between these studies may arise from differences in (i) the methods of assessing hearing loss, and/or (ii) the OI populations (type, age, gender) examined, and/or (iii) clinical assessments of OI type: observational in the past and based on genetic analysis in the last decade.

The mechanisms of hearing loss in OI are still unclear. OI’s most affected anatomical structures include the otic capsule, oval and round windows, stapediovestibular joint, and internal auditory canals^17,25–29^. Common manifestations of conductive hearing loss in OI include a fixed and/or thick stapes footplate^5,30–47^, associated with microcracks accumulation of unclear origin, which may relate to the increased porosity and brittleness of the OI bone^48^, and that lead to localized bone overgrowth^49^. Ossicular discontinuity, mainly caused by fractures in the stapes crura, is commonly reported in cases of conductive hearing loss in OI^31,33–36,38–42,46,47,50,51^. Manifestations of sensorineural hearing loss in OI include hyalinization and atrophy of the stria vascularis, hair cell atrophy, and microfractures of the cochlear otic capsule^17,20^. Documented otologic microsurgeries in some OI individuals also revealed tectorial membrane distortion and perilymph hemorrhage^52^. In other OI cases, sclerotic thickening of the cochlear otic capsule and a brittle scutum has been reported^53^, and demineralization of the bone around the cochlea and vestibule and partial ossification of the basal turn of the cochlea^54^. In OI, mixed hearing loss often arises with fenestral involvement causing conductive hearing loss and progresses toward the retrofenestral components with age, causing sensorineural hearing loss^6,8,12,21,24^. Interestingly, Swinnen et al.^49^ found an association between whole body low bone mineral density and conductive or mixed hearing loss in people with OI. The OI population suffering from hearing loss also have elevated alkaline phosphatase levels^27^, an enzyme recently reported to be associated with hearing loss in the general population and suggested to be critical for the diagnosis and progression assessment of auditory impairments^55^.

Although middle ear lesions reported in OI are similar to those reported in patients with otosclerosis, hearing loss in OI is different: it arises in childhood and is characterized by ossicle atrophies or fractures, and high porosity, and a thicker otic capsule^39,40^. Otosclerosis is limited to the otic capsule’s endochondral layer, whereas OI involves the endosteum, endochondral layer, and periosteum^29^. Despite these differences, in the last decades, treatment strategies for hearing loss in OI have been the same as these for the general population's auditory impairments. However, it is unknown how successful they are in the OI population, a disease with no cure. Therefore, this study aims to assess current treatments’ efficacy addressing hearing loss in OI, employing a systematic review and a meta-analysis.

## Methods

### A systematic review on the efficacy of treatments for hearing loss in the OI population

This study conforms to reporting standards of the Preferred Reporting Items for Systematic Reviews and Meta-Analyses (PRISMA). A systematic review was conducted on the hearing restoration in OI patients after stapes surgeries, cochlear implantation, hearing restoration with bone-anchored hearing aids (BAHA), implantable/non-implantable hearing aids, and drug treatments. The intended population, intervention, comparator, outcome, timing, and settings of this study are summarized in a PICOT table (Table 1). This study’s data sources include Medline via PubMed, Scopus, Web of Science, and Embase, from their inception to November 2020. The search consisted of studies that included hearing loss treatments in people with OI. Search terms related to hearing loss treatments in OI were combined to optimize the results. Keywords included *hearing loss*, *treatment*, *osteogenesis imperfecta, stapes surgery*, *stapedectomy*, *stapedotomy*, *cochlear implant*, *hearing aid*, *drug, bisphosphonates*. No language restriction was applied. Reference lists and citations were reviewed to include studies that were missed in the original search.

**Table 1:** Population, intervention, comparison, outcome, timing and setting (PICOTS) of the therapies to ameliorate hearing loss in osteogenesis imperfecta (OI).

Key question	How successful are current therapies to ameliorate hearing loss in the OI population?
Population	OI-subjects with hearing loss
Intervention	Internal and external implants, and drug therapies
Comparison	Pre- and post-operative hearing function in the same patient measured by audiograms or speech perception scores
Outcome	Proportion of the cohort with amelioration in the post-operative hearing, or patient-specific post-operative results
Timing	According to reports in the trials (most are within a 12-months period post-operative follow-up)
Setting	To inform considerations for the choice and timing of intervention in the OI population

#### Eligibility criteria

We included publications that addressed hearing loss treatments on individuals with OI and quantitatively and objectively assessed hearing loss before and after treatment at specified time points and follow-up lengths. The lack of auditory assessment, no specified length of follow-up, averaged results for all the cohort of people considered, and mixed data for general and the OI populations were reasons for exclusion of the study. Reports counting the same patients twice were also excluded. Furthermore, because this study aims to determine the success of the primary intervention, studies reporting on revision cases for more than 15% of their cohort were excluded. Any article deviating from the objective of this study was rejected.

#### Screening and data extraction

Two authors searched and screened the included studies, and records were noted in an excel sheet. Duplicated studies were identified and removed. Consequently, abstracts were reviewed, and those deviated from the study’s objective were excluded. The remaining manuscripts were reviewed in full text and included in the study if they complied the inclusion criteria. Disagreements among researchers were resolved through consensus or third-party adjudication. Data about treatment strategy, population characteristics, outcomes of interest, and length of follow-up were extracted. One person performed data collection while a second person reviewed the extracted data. Data was then analyzed and divided to conduct: (1) a meta-analysis on the efficacy of stapes surgery in OI and (2) a systematic review on the rest of the treatments for hearing loss in OI. The studies included in the systematic review were single study cases with 1–3 individuals. They did not allow for a meta-analysis to be conducted due to the small total number of individuals considered and the lack of data about treatment efficacy*.*

#### Quality assessment

The quality of the articles included in this meta-analysis and systematic review was determined by following three quality assessment tools from The National Institutes of Health^56^, according to the analyzed type of study. Namely, the NIH quality assessment tools for (1) Before-After (Pre-Post) Studies With No Control Group were used for the studies in the meta-analysis; (2) Case Series studies were used for the studies in the systematic review; (3) Observational Cohort and Cross-Sectional Studies were used for the two observational studies reporting on the effect of bisphosphonates on hearing function^13,57^ as in their cohort bisphosphonate treatments were not intended as a first treatment for hearing loss in OI.

### Meta-analysis for the efficacy of stapes surgery in OI

#### Inclusion criteria

Inclusion criteria comprised published studies on people with OI who have been diagnosed with hearing loss using pure tone audiometry and underwent primary stapes surgery (unilateral or bilateral) and have been followed up in the clinic at specified times. Excluded publications were articles without information on stapes intervention, studies without pure tone audiometry results, not reporting postoperative audiometry outcomes, articles performing revision surgeries in > 15% of their cohort, studies presenting solely graphical data, and reporting mean values for the entire cohort. Studies that reported the Air–Bone Gap (ABG) value as a mean for the entire cohort rather than patient-specific ABG values or the percentage of the subjects with an ABG ≤ 10 dB, were also excluded from the selection.

#### Screening and data extraction

According to the aforementioned screening process, data was extracted from the selected articles for meta-analysis. The database included article characteristics (i.e., authors, title, and year of publication), treatment strategy (i.e., stapes surgery), pure-tone audiometry results (i.e., preoperative and postoperative results at the short term, ≤ 12 months), population characteristics (i.e., mean age at primary surgery, and the number of ears undergoing treatment) and length of follow-up. The level of evidence of each study included in the meta-analysis was evaluated through Oxford Centre for Evidence-Based Medicine criteria^58^.

#### Statistical analysis

Statistic calculus and analysis were conducted using MedCalc 19.2.3 (MedCalc Software Ltd, Ostend, Belgium; https://www.medcalc.org; 2020), and a proportion meta-analysis was conducted on the efficacy of stapes surgeries. Because closure of the ABG within 10 dB or less is considered a successful outcome of stapes surgeries in the literature^37,46,59^, the efficacy of stapes surgeries was quantified as the proportion of ears having an ABG ≤ 10 dB. An effect size (proportion of successful cases) was calculated for each study with its corresponding 95% confidence interval (CI).

Because of the low power of meta-analyses with a small number of studies, as in this case, we assessed the heterogeneity between studies with Cochran’s Q test, which low P-value indicated heterogeneity. Specifically, we reported the inconsistency (I), quantified using ${I}^{2}$ statistic, which describes the percentage of variability in pooled effects caused by heterogeneity rather than chance^43^. ${I}^{2}$ ≥ 50 and P < 0.1 were considered for significant heterogeneity, as suggested by Higgins et al.^60^. Thus a random-effects model was tested instead of a fixed-effects model in the meta-analysis.

#### Risk of bias within the meta-analysis

Egger’s test^61^ and Begg’s rank test^62^ were used to detect possible publication bias using MedCalc. A low P-value (< 0.05) indicates publication bias in both tests. Funnel plots were also created to detect bias in the meta-analysis. Asymmetry of the funnel plots was evaluated considering that heterogeneity factors could have an effect^63^.

## Results

**Figure 1:**
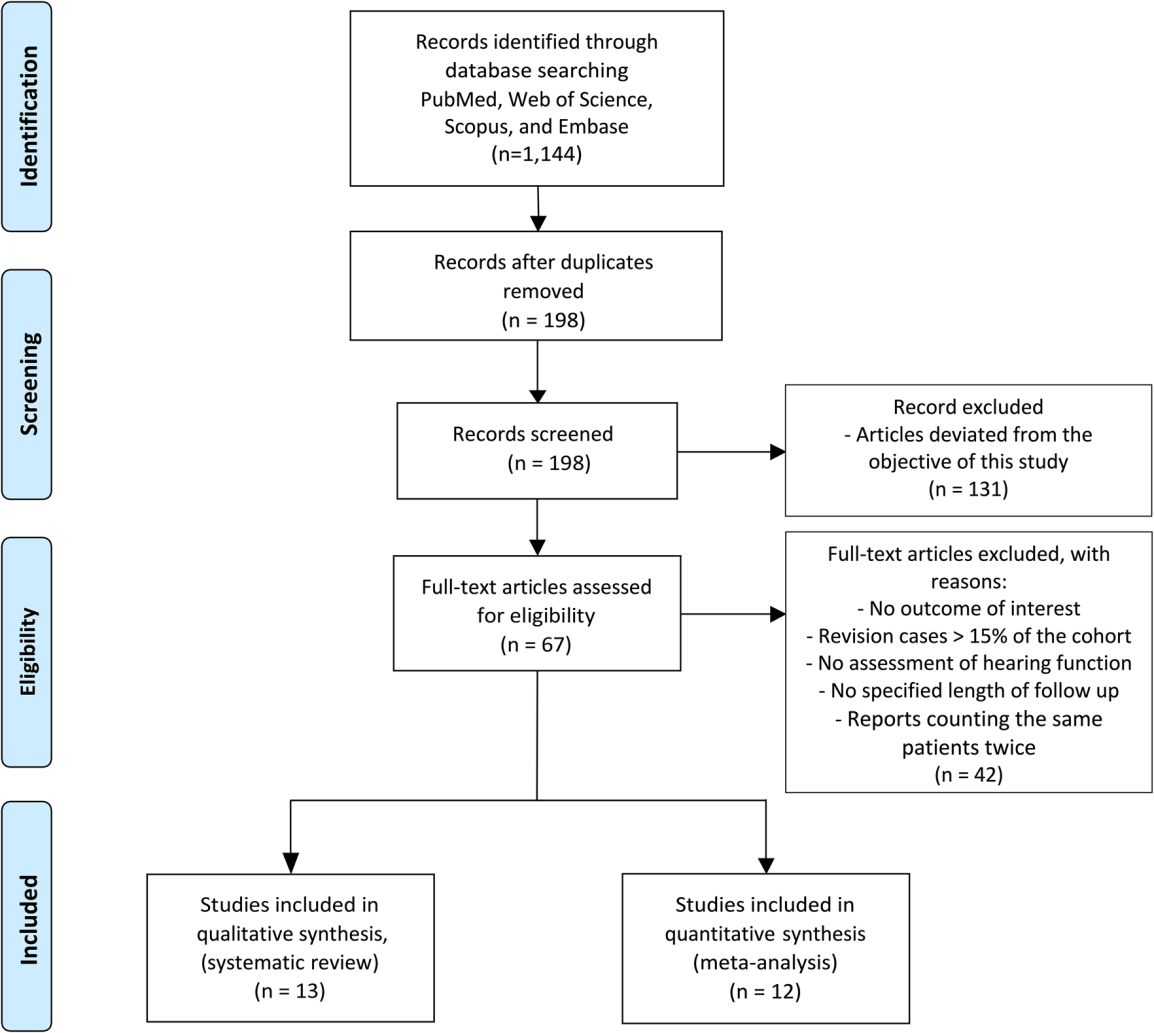
PRISMA flow diagram. A total of 1144 articles were retrieved from the search process. After removing duplicates, 198 studies were screened at the abstract level. After removing articles (n = 131) that deviated from the objective of this study, 67 articles were reviewed at full text. 42 articles did not satisfy the inclusion criteria and were excluded from subsequent screening. Among the articles left, 13 met the inclusion criteria for the systematic review, and 12 different studies met the inclusion criteria for the meta-analysis.

The literature search identified 1144 studies, of which 946 duplicates were excluded. Among the remaining 198 studies, 131 were excluded at the abstract level, and 67 articles were reviewed at full text. Twelve articles met the inclusion criteria and were included for meta-analysis, whereas 13 were included for systematic review (Fig. 1).

### A meta-analysis on the efficacy of stapes surgery in the OI population

The quality assessment of the articles included in the meta-analysis is reported in Supplementary Table S1. All studies except three were rated as good quality studies; the remaining three articles were rated as fair quality studies because they poorly described the intervention. Data extraction and collection was performed on the 12 articles that met the inclusion criteria for meta-analysis (Table 2). The level of evidence of each article was assessed as IV, referring to well-designed case-series or cohort studies^58^. The proportion of ears having an ABG ≤ 10 dB was extracted as the primary outcome of interest from each paper. We also extracted and collected the proportion of ears with a 10 < ABG < 20 dB and ABG > 20 dB, mean ABG value, length of follow-up, number of ears undergoing stapes surgery, and mean age at primary surgery. Both short-term (≤ 12 months) and long-term (> 12 months) audiometry values were extracted. Intraoperative findings encountered in these 12 reports are reported in Table 3 and include multiple cases of fixed and/or thick stapes footplate, followed by cases of vascular mucosa, thin or/and atrophic stapes crura, and in less frequency fractured stapes crura.

**Table 2:** Short-term and long-term audiometric postoperative results following stapes surgery in individuals with osteogenesis imperfecta.

Study	Level of evidence	Mean age at primary surgery (range)	Frequency range measured	Short term (≤ 12 months) results					
				Long term (> 12 months) results					
				No. of ears	Mean follow up (months)	ABG ≤ 10 dB, no. of ears (%)	10 < ABG < 20 dB, no. of ears (%)	ABG > 20 dB, no. of ears (%)	Mean ABG, dB
Shea and Postma (1982)^40^	4	–	0.5, 1.0 and 2.0 kHz	51	12	38 (75)	–	–	12
				24	84	–	–	–	–
Pedersen (1983)^76^	4	31	0.5, 1.0, 2.0, and 4.0 kHz	42	3	26 (62)	9 (21)	7 (17)	–
				–	–	–	–	–	–
Garretsen and Cremers (1990)^43^	4	30.6	0.5, 1.0 and 2.0 kHz	52	3	37 (71)	11 (21)	4 (8)	10
				37	115	26 (70)	7 (19)	4 (11)	12
Albahnasawy et al. (2001)^46^	4	36.4 (25–55)	0.5, 1.0, 2.0, and 4.0 kHz	5	6–12	4 (80)	1 (20)	0 (0)	8
				–	–	–	–	–	–
Van der Rjit and Cremers (2003)^47^	4	38	0.5, 1.0 and 2.0 kHz	13	1.5	4 (31)	7 (50)	3 (21)	–
				–	–	–	–	–	–
Kuurila et al. (2004)^31^	4	30.1 (15–53)	0.5, 1.0 and 2.0 kHz	43	6–12	18 (42)	13 (30)	12 (28)	–
				–	–	–	–	–	–
Vincent et al. (2005)^32^	4	37 (18–67)	0.5, 1.0, 2.0, and 4.0 kHz	21	1.5	18 (86)	0 (0)	3 (14)	4
				10	24	8 (80)	–	–	5.6
Swinnen et al. (2009)^34^	4	40 (17–71)	0.5, 1.0, 2.0 and 3.0 kHz	12	1.5	8 (67)	4 (33)	0 (0)	8
				8	42	6 (75)	1 (13)	1(13)	9
Swinnen et al. (2012)^35^	4	32.7 (13–69)	0.5, 1.0, 2.0 and 3.0 kHz	28	2	17 (61)	10 (36)	1 (3)	8.2
				18	168	18 (100)	0 (0)	0 (0)	4.8
Vincent et al. (2014)^37^	4	36 (18–59)	0.5, 1.0, 2.0, and 4.0 kHz	32	4	28 (88)	–	–	4.3
				18	26	13 (72)	–	–	4.7
Skarzyński et al. (2019)^38^	4	32.5 (14–63)	0.5, 1.0, 2.0, and 4.0 kHz	21	–	9 (43)	–	–	12.26
				17	–	7 (41)	–	–	11.54
Ma et al. (2020)^39^	4	28.5 (12–48)	0.5, 1.0, 2.0 and 3.0 kHz	17	–	1 (6)	8 (47)	8 (47)	19
				10	36	3 (30)	3 (30)	4 (40)	20

No sufficient data is indicated with a line (-). *ABG* is Air Bone Gap. Level of evidence 4 refers to well-designed case-series or cohort studies^58^.

**Table 3:** Middle ear complications in individuals with osteogenesis imperfecta that underwent stapes surgery.

Study	No. of ears	Fixed stapes footplate No. of ears	Thick stapes footplate No. of ears	Fractured stapes crura No. of ears	Thin or/and atrophic stapes crura No. of ears	Vascular mucosa No. of ears
Shea and Postma (1982)^40^	62	62	31	–	13	18
Pedersen (1983)^76^	43	43	23	5	14	13
Garretsen and Cremers (1991)^51^	58	54	32	10	22	12
Albahnasawy et al. (2001)^46^	6	3	–	4	–	–
Van der Rijt and Cremers (2003)^47^	13	13	7	1	9	6
Kuurila et al. (2004)^31^	43	–	19	4	4	16
Vincent et al. (2005)^32^	23	23	–	0	0	–
Swinnen et al. (2009)^34^	13	13	4	1	4	2
Swinnen et al. (2012)^35^	29	29	22	1	13	6
Vincent et al. (2014)^37^	32	32	32	0	0	–
Skarzynski et al. (2019)^38^	24	13	6	4	–	9
Ma et al. (2020)^39^	22	22	12	7	3	10

Fixed and thick stapes footplates, crura fractures, thin and atrophic stapes, and vascular mucosa were among the most complications reported during stapes surgery in the OI population.

**Figure 2:**
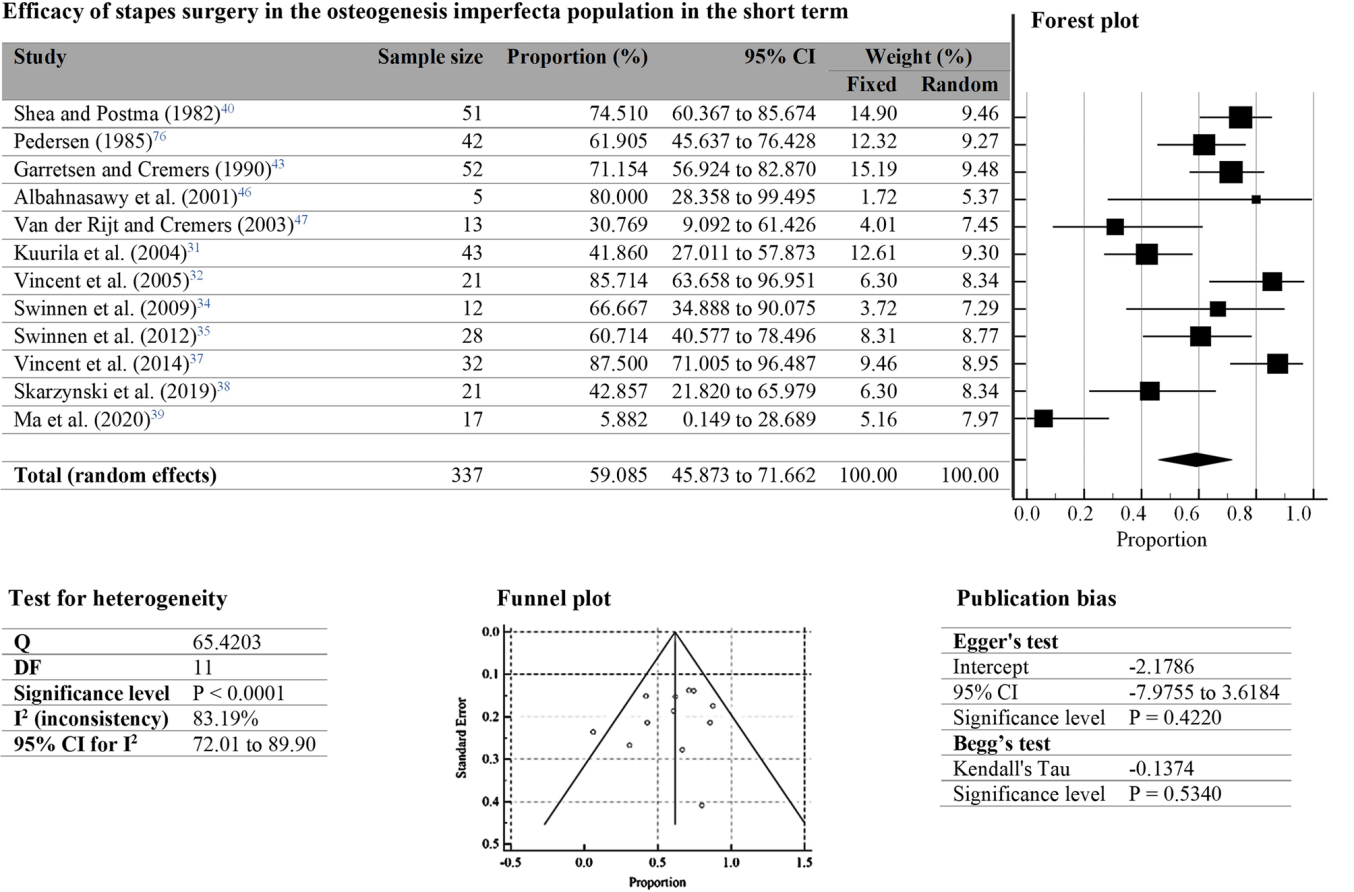
Efficacy of stapes surgery in the osteogenesis imperfecta population in the short term. Studies included for meta-analysis are shown in the table together with the overall result with its corresponding sample size, effect size represented as a proportion (proportion of ears with a postoperative Air Bone Gap (ABG) ≤ 10 dB), 95% confidence interval (CI), and weight for random effect model. The forest plot graphically represents the results. There, each study is represented with a box. The size of each box represents the weight of each study whereas the lines determine their 95% CI. The overall result is shown by a diamond in which the middle indicates the estimate, and the sides represent its 95% CI. Our meta-analysis shows an overall result of 59.01% (95% CI 45.87 to 71.66). The test for heterogeneity table shows Cochrane’s Q test, degrees of freedom (DF), significance level, inconsistency value (${I}^{2}$), and its corresponding 95% CI. Significative heterogeneity was found in our results (P < 0.0001) and ${I}^{2}$ of 83.19% (95% CI 72.01 to 89.9). The funnel plot was used to detect publication bias of meta-analysis by plotting the standard error over the proportion (effect size). The diagonal lines represent the 95% CI around the summary effect size. Studies are represented with dots. Publication bias were also assessed with Egger’s and Begg’s test, represented with an intercept, a 95% CI and a significance level; and Kendall’s Tau and significance level, respectively. Results indicate low to no risk of bias across studies (Egger’s test, P = 0.42; and Begg’s test, P = 0.53).

Twelve studies assessed the outcomes of primary stapes surgeries using pure-tone audiometry in individuals with OI. All of them included the proportion of ears showing an ABG ≤ 10 dB and length of follow-up. Long-term results showed an evident reduction in individuals’ participation during follow-up, introducing biases for those measurements. Thus, we only considered short-term effects (within 12 months) for the meta-analysis, and a random-effects model meta-analysis was conducted. With a total sample size of 337 cases within the 12 manuscripts here considered, our results show that stapes surgeries have a short-term success rate of 59.08 (95% CI 45.87 to 71.66) in the OI population (Fig. 2).

The test for heterogeneity showed a low P-value (P < 0.0001) and ${I}^{2}$ of 83.19% (95% CI 72.01 to 89.90), indicating significant heterogeneity across studies. In eight of the included studies, the proportion of ears showing an ABG ≤ 10 dB was higher than 60%. Three of the remaining studies reported success of ≥ 31%, except for Ma et al.’s study^39^, which reported that only 5.88% of patients obtained a postoperative ABG ≤ 10 dB, thus showing results substantially different from the rest of the included studies. Ma et al. attributed the difference to several factors: first, they encountered a higher rate of stapes crura fractures than that reported in the rest of included studies; second, they performed one surgery with laser, which was a relatively new technique for the operating surgeon; third, a high number of cases with mucosal bleeding were reported and the authors claimed that their OI cases were more severe than those included in the other manuscripts; and finally, the use of fat instead of a prosthesis in an ear could have also affected their results^39^.

Regarding the risk of bias within the meta-analysis, Both Egger’s test and Begg’s test show high P-values (P = 0.422 and P = 0.534, respectively), indicating low to no risk of bias across studies. Also, the symmetry of the funnel plots in Fig. 2 well represented little to no publication bias.

### Efficacy of other treatments for hearing loss in the OI population

The quality assessment of the studies included for systematic review can be found in Tables 4 and 5. All studies except one were rated as good quality studies. The remaining article had a fair quality due to poor definition of the outcome measures and inadequate follow-up.

**Table 4:** Quality assessment tool for case-series studies of the studies included in the systematic review.

NIH quality assessment tool for Case Series studies	Effect of cochlear implantations on hearing loss in osteogenesis imperfecta										
	Szilvássy et al. (1998)^67^	Huang et al. (1998)^77^	Migirov et al. (2003)^64^	Streubel and Lustig (2005)^27^	Rotteveel et al. (2008)^53^	Sainz et al. (2009)^65^	Heo et al. (2009)^78^	Makizumi et al. (2013)^66^	Marfatia et al. (2020)^54^	Coutinho et al. (2015)^68^	Kontorinis et al. (2011)^69^
1. Was the study question or objective clearly stated?	Yes	Yes	Yes	Yes	Yes	Yes	Yes	Yes	Yes	Yes	Yes
2. Was the study population clearly and fully described, including a case definition?	Yes	Yes	Yes	Yes	Yes	Yes	Yes	Yes	No	Yes	Yes
3. Were the cases consecutive?	NA	NA	NA	NA	NA	NA	NA	NA	NA	NA	NA
4. Were the subjects comparable?	NA	NA	NA	Yes	Yes	NA	NA	NA	NA	NA	NA
5. Was the intervention clearly described?	Yes	Yes	Yes	Yes	Yes	Yes	Yes	Yes	Yes	Yes	Yes
6. Were the outcome measures clearly defined, valid, reliable, and implemented consistently across all study participants?	No	Yes	Yes	Yes	Yes	Yes	Yes	Yes	Yes	Yes	Yes
7. Was the length of follow-up adequate?	No	Yes	Yes	Yes	Yes	Yes	Yes	Yes	Yes	Yes	Yes
8. Were the statistical methods well-described?	NA	NA	NA	NA	NA	NA	NA	NA	NA	NA	NA
9. Were the results well-described?	Yes	Yes	Yes	Yes	Yes	Yes	Yes	Yes	Yes	Yes	Yes

*NA* indicates not applicable.

**Table 5:** Quality assessment tool for observational cohort and cross-sectional studies of the studies reporting on the effect of bisphosphonate on hearing loss in adults^57^, and children^13^ with osteogenesis imperfecta.

NIH quality assessment tool for observational cohort and cross-sectional studies	Effect of bisphosphonates on hearing loss in osteogenesis imperfecta	
	Adults—Brodd et al. (2011)^57^	Children—Ting et al. (2012)^13^
1. Was the research question or objective in this paper clearly stated?	Yes	Yes
2. Was the study population clearly specified and defined?	Yes	Yes
3. Was the participation rate of eligible persons at least 50%?	NA	NA
4. Were all the subjects selected or recruited from the same or similar populations (including the same time period)? Were inclusion and exclusion criteria for being in the study prespecified and applied uniformly to all participants?	Yes	Yes
5. Was a sample size justification, power description, or variance and effect estimates provided?	No	No
6. For the analyses in this paper, were the exposure(s) of interest measured prior to the outcome(s) being measured?	Yes	Yes
7. Was the timeframe sufficient so that one could reasonably expect to see an association between exposure and outcome if it existed?	Yes	Yes
8. For exposures that can vary in amount or level, did the study examine different levels of the exposure as related to the outcome (e.g., categories of exposure, or exposure measured as continuous variable)?	Yes	Yes
9. Were the exposure measures (independent variables) clearly defined, valid, reliable, and implemented consistently across all study participants?	NA	NA
10. Was the exposure(s) assessed more than once over time?	NA	NA
11. Were the outcome measures (dependent variables) clearly defined, valid, reliable, and implemented consistently across all study participants?	Yes	Yes
12. Were the outcome assessors blinded to the exposure status of participants?	No	No
13. Was loss to follow-up after baseline 20% or less?	Yes	Yes
14. Were key potential confounding variables measured and adjusted statistically for their impact on the relationship between exposure(s) and outcome(s)?	No	No

*NA* indicates not applicable.

#### Cochlear implants

A total of nine studies reported outcomes on 11 cases of unilateral cochlear implantations in OI individuals. Table 6 reports each study’s results corresponding to their latest follow-up in the short-term. In all cases, the cochlear implant recipients were adults, except for the cases reported by Migirov et al.^64^ and Marfatia et al.^54^, in which the individuals were 6 and 14 years old, respectively, at the time of surgery.

**Table 6:** Short-term (< 12 months) outcomes of cochlear implantations in individuals with osteogenesis imperfecta (OI).

Study	Age at surgery	OI type	Sex	Preoperative speech perception	Postoperative speech perception	Follow-up
Szilvássy et al. (1998)^67^	50	–	F	Wearing a hearing aid closed set speech recognition test revealed invaluably low results	“Significant improvement in hearing”	7 days
Huang et al. (1998)^77^	42	–	F	Wearing a hearing aid: - Word: 12% - Sentence: 24% - Vowel: 60%	- Word: 44% - Sentence: 59% - Vowel: 94%	3 months
Migirov et al. (2003)^64^	6	–	M	Some words identified	OS word identification: - Mono-syllabic words: 25% - Two-syllable words: 40% - Hebrew speech pattern contrast test: 44%	6 months
Streubel and Lustig (2005)^27^	35	Type I	F	- Phoneme score (CNC): 0% - Words score (CNC): 0% - Sentence score (CID): 0%	- Phoneme score (CNC): 75% - Words score (CNC): 54% - Sentence score (CID): 99%	12 months
	–	Type I	F	- Phoneme score (CNC): 12% - Words score (CNC): 4% - Sentence score (CID): 75%	- Phoneme score (CNC): 83% - Words (CNC): 70% - Sentence score (CID): 100%	12 months
Rotteveel et al. (2008)^53^	45	Type I	F	–	- Phoneme score: 84% - Word score: 60% NVA-test	12 months
	51	Type I	F	–	- Phoneme score: 78% - Word score: 56% NVA-test	12 months
Sainz et al. (2009)^65^	–	Type I		–	The electrode guide insertion was arrested and deviated multiple times while attempting the implantation that was averted	–
Heo et al. (2009)^78^	39	–	F	- CS 1-syllable word identification: 0% - OS 1-syllable word identification: 0% - Length of words identification: 8.3% - Word comprehension: 43.55% - Sentence comprehension: 30%	- CS 1-syllable word identification: 100% - OS 1-syllable word identification: 100% - Length of words identification: 100% - Word comprehension: 100% - Sentence comprehension: 100%	6 months
Makizumi et al. (2013)^66^	52	–	F	Wearing a hearing aid: Phonemes 10%	- Monosyllables: 62% - Words 79% - Sentences: 91%	6 months
Marfatia et al. (2020)^54^	14	–	F	–	Word recognition score 45% at 60 dB	12 months

No sufficient data is represented with a line (–).

*F* indicates female, *M* male, *OS* open-set, *CNC* consonant noun consonant, *CID* The Central Institute for the Deaf, *NVA* Dutch Audiological Society, and *CS* closed-set.

Among the above-identified articles, cochlear implantation in people with OI showed improvement in speech perception scores in the short term (≤ 12 months) in 10 out of 11 reported cases. A non-successful outcome was related to a placement failure of the electrode array^65^. In all cases, the implantation was challenging because of ear hypervascularization, severe demineralization of the otic capsule, proliferation of the round window niche, and lack of anatomical landmarks typical of the OI population^27,53,54,66^. A high incidence of facial nerve stimulation after cochlear implantation was also common in people with OI^27,53,54,64,66,67^, and in three cases it was controlled through programming strategies^27,53,67^.

#### Bone anchored hearing aids

Only one study described the efficacy of BAHA in an individual (aged 45) suffering from OI type III hearing loss in a single ear^68^. Despite the brittleness of the OI bone, this single case was successful with free field audiometry showing a mean improvement of about 45 dB (at frequencies: 0.25, 0.5, 1, 2, and 4 kHz). Improvement was also observed in the speech audiometry results when fitting the bone-anchored hearing aid, with approximately a 40 dB hearing function difference. Implant osteointegration was reported, given by implant stability quotient values measured by resonance frequency analysis in four cardinal points of the abutment^68^.

#### Implantable hearing aids

Sainz et al.^65^ reported no benefit in a person with OI type I fitted with an implantable hearing aid, in which the transducer was placed at the round window. Kontorinis et al.^69^, on the other hand, reported three successful cases in two people with OI type I, fitted with implantable hearing aids. In these two people, the transducer was attached to the long process of the incus in the proximity of the incudostapedial joint, and stapedotomy was performed. They reported a mean preoperative bone conduction and air-conduction threshold of 47.1 dB (range 40–55 dB) and 79 dB (range 60–90 dB), respectively, and postoperative bone conduction and air conduction threshold of 45 dB (range 36.3–50 dB) and 42.1 dB (range 32.5–51.3 dB), respectively.

#### Hearing aids

Hearing aids are widely used in the OI population until they show no longer benefit caused by the progressing hearing loss^8,22,31,54,70^. However, no study was found reporting data about their efficacy.

#### Bisphosphonates effects on hearing loss

Two studies reported the effects of bisphosphonates on the hearing function in OI^13,57^. The study of Ting and Zacharin^13^ included children treated with either pamidronate (oral 1 mg/kg 2 monthly) or zoledronic acid (intravenous 0.05 mg/kg 4 monthly), for at least 2 years. Hearing function was assessed using tympanometry and pure tone audiometry at 0.5, 1, 2, and 4 kHz^13^. The study by Brodd et al.^57^ included adults with OI who received either intravenous injections of pamidronate or weekly oral alendronate tablets and supplements of calcium and vitamin D for at least 3 years. Pure tone audiometry at 0.5, 1, 2, 3 and 4 kHz was conducted to assess hearing^57^. The results showed that bisphosphonates have non-significant effects on the hearing of adults with OI. In contrast, Ting and Zacharin^13^ reported that the incidence of hearing loss was substantially lower in children treated with bisphosphonates for their bone fragility, compared to the untreated subjects from their previous studies. The authors^13^ suggested that treatment with bisphosphonates might reduce or halt the natural progression of hearing loss in OI. The limitation of their study is that they only assessed hearing in 4 of their 36 patients after treatment^13^.

## Discussion

This study examined the efficacy of current treatment strategies to ameliorate hearing loss in OI. Our results show that treatments addressing hearing loss in OI rely mostly on conventional treatments for auditory impairments in the general population. Together with hearing aids and cochlear implants, stapes implants are widely used in OI. Nevertheless, their efficacy is limited, and their success rate is impacted by the bone fragility and high vascularity typical of the disease. Rare is the use of BAHA and implantable hearing aids in OI^25,68,69^. Finally, drug treatments for hearing loss in OI are not yet used. The lack of knowledge on the disease mechanisms and progress affecting the inner and middle ear disfunction in OI plays a major role on the absence of suitable treatments for hearing loss in OI.

Middle ear surgery is currently the treatment of choice for conductive hearing loss in OI when hearing aids are no longer beneficial. However, our meta-analysis shows that stapes surgery has a low 59.08% success rate in the OI population. Disease related changes of middle ears typically found in patients with OI, including atrophies or fractures of the ossicles^31,34–36,39,41–43,47,50^ make surgeries challenging for the OI population^42^ and likely to require revisions^71^. Our meta-analysis heterogeneity values showed that the proportion of ears having an ABG ≤ 10 dB is a highly variable effect across studies with people with OI. Factors influencing this variation could not be determined here. Still, they can be attributed to the different OI types included in the cohort of each study, age and gender of the individuals involved, and the ability of the surgeon performing the interventions. The variability in outcome among the studies considered here may also be attributed to absence of the definition of hearing loss in the included studies. For our meta-analysis we assumed that studies defined hearing loss as thresholds elevations of 15 dB or 20 dB^5,7,8,35,51^. A previous study reported similar outcomes and intraoperative findings for different OI types^35^. However, we could not assess these variables’ effect due to a lack of reported data. Hence, we suggest that studies publish detailed data and distinguished outcomes for different OI types. Inconsistency of follow-up time was observed across studies. Here, we considered only a follow-up time that was in the range of months for all the studies, so that this factor has little to no effect on the meta-analysis results. For stapes surgery, small deterioration of the postoperative outcomes is expected over the years^31,32,35,37,51^, and our comparisons are made in a range of months. Furthermore, the loss of follow-up in some of the stapes surgery studies in OI limited our study to the use of only outcomes for short-term follow-up. Long-term follow-up and data on revision could give a better prospect on the actual duration of the efficacy of stapes surgery in OI. According to Skarżyński et al.^71^, stapes surgeries in OI have an elevated risk of requiring revision surgeries. The brittle nature of OI bone and common intraoperative complications reported in the literature for stapes surgery in individuals with OI (Table 3) indicate stapes surgery as a risky surgical procedure.

A potential limitation of this meta-analysis and systematic review is intrinsic to the small number of reported studies of hearing loss in OI subjects, that is related to the rarity of the disease. To account for the highest number of studies in conducting this meta-analysis, we included studies over a 40 year time span, which is, however, justified by the very little changes in the stapes surgery approach over this period of time. The results from the included studies showed no trend associated with the chosen time frame. However, variability was observed in studies conducted within the same few years (Forest plot, Fig. 2). The included studies in the meta-analysis were classified as studies of level IV of evidence. Although meta-analyses are often conducted on studies with higher levels of evidence, a meta-analysis of well-designed case-series that is based on a comprehensive search strategy is a valid and useful statistic tool to utilize in the absence of clinical trials^72^. A proportional meta-analysis on case series is intended to aid clinicians and patients in their decisions until higher quality studies are performed.

The systematic review conducted on the rest of the treatments addressing hearing loss in OI showed that cochlear implants, BAHA, and implantable hearing aids proved to be feasible in people with OI, in general, with successful results in the very few reported cases. The number of published articles on cochlear implantations in OI is limited. Cochlear implantation proved mostly feasible and successful in ameliorating hearing loss in individuals with OI in the handful of published single cases (10/11). Challenges and complications were reported associated with the insertion of the electrode array in individuals with OI^65,73^ due to hypervascularity of the pericochlear bone and excessive bone growth^53^, or difficulties in identifying the round or oval window niche because of excessive bone formation in these areas^27^. Facial nerve stimulation in OI subsequent to cochlear implantations has been suggested to result from a decreased electrical resistance of the temporal bone and the thin bone separating the Fallopian canal from the cochlea, and can be solved by switching off the channels stimulating the facial nerve^67^.

The single case reporting on the BAHA implant in a person with OI claims that OI’s characteristic brittle bone is not a constraint for selected cases. Swinnen et al.^8^, also reported the use of BAHA in people with OI, but no data was reported about their efficacy. Implantable hearing aids have shown good outcomes in the few reported cases and might serve as a solution for hearing loss in the OI population. Anyhow, further studies are needed to strengthen the results on its efficacy and feasibility. No study was found on the efficacy of hearing aids in the OI population, although they are widely used due to their cheap cost.

Recently, bisphosphonates, a group of antiresorptive drugs for bone and the standard-of-care treatment for severe OI, have been suggested to be a treatment of hearing loss by stabilizing sensorineural hearing loss in cochlear otosclerosis^74,75^. While bisphosphonates are used to increase bone quantity in OI, it is not clear whether they also reduce hearing loss. Because of their wide use in the OI population, future studies should investigate their effect on the audiological performances of people with OI.

The lack of knowledge of the mechanisms inducing hearing loss in OI constitutes a major limitation to the use of effective treatments. Clinical observational studies are needed to further understand the evolution of hearing loss in the OI population. Furthermore, preclinical studies using animal models of OI suffering from hearing loss are required to understand the etiology and mechanisms of hearing loss, and to develop new targeted treatments to prevent auditory impairments in OI. Hearing function as well as ear biomechanics and quality of collagenous tissues at multiple length scales should be investigated with multidisciplinary approaches. Therapies for hearing loss in OI should be further investigated for their direct and side effects in animal models. Finally, clinical trials can be conducted to investigate efficacy of a single or combination of treatments, for both hearing loss and bone fragility, in the OI population.

## Conclusions

This systematic review and meta-analysis on the current treatments of hearing loss in OI shows that the efficacy of stapes surgeries has a low 59.08% success rate, and the other treatments addressing hearing loss in OI (i.e., cochlear implants, BAHA, and implantable hearing aids) showed to be feasible and successful in ameliorating hearing loss in the OI population in the very few reported single cases. This study emphasizes the need of further research to understand the mechanism(s) of OI leading to hearing loss and to advance current and new treatment strategies to prevent or reduce hearing loss in OI.

## Supplementary Information

Supplementary Table S1.

## Data Availability

All data analyzed in this systematic review and meta-analysis has been published in the literature before and their references are included in this published article (and its Supplementary Information files).
