# Supplementary material for: Treatments for hearing loss in osteogenesis imperfecta: a systematic review and meta-analysis on their efficacy
Source: Sci Rep. 2022 Oct 12;12:17125. doi: 10.1038/s41598-022-20169-9 (PMC9556526; doi:10.1038/s41598-022-20169-9)
Supplement: Supplementary file 1 — Supplementary Table S1. [file 41598_2022_20169_MOESM1_ESM.docx]

| **NIH quality assessment tool for Before-After (Pre-Post) Studies With No Control Group** | Shea and Postma, 1982 [1] | Pedersen, 1983 [2] | Garretsen and Cremers, 1990 [3] | Albahnasawy et al., 2001 [4] | Van der Rjit and Cremers, 2003 [5] | Kuurila et al., 2004 [6] | Vincent et al., 2005 [7] | Swinnen et al., 2009 [8] | Swinnen et al., 2012 [9] | Vincent et al., 2014 [10] | Skarzyński et al., 2019 [11] | Ma et al., 2020 [12] |
| --- | --- | --- | --- | --- | --- | --- | --- | --- | --- | --- | --- | --- |
| 1. Was the study question or objective clearly stated? | Yes | Yes | Yes | Yes | Yes | Yes | Yes | Yes | Yes | Yes | Yes | Yes |
| 2. Were eligibility/selection criteria for the study population prespecified and clearly described? | NA | NA | NA | NA | NA | NA | NA | NA | NA | NA | NA | NA |
| 3. Were the participants in the study representative of those who would be eligible for the test/service/intervention in the general or clinical population of interest? | Yes | Yes | Yes | Yes | Yes | Yes | Yes | Yes | Yes | Yes | Yes | Yes |
| 4. Were all eligible participants that met the prespecified entry criteria enrolled? | NA | NA | NA | NA | NA | NA | NA | NA | NA | NA | NA | NA |
| 5. Was the sample size sufficiently large to provide confidence in the findings? | Yes | Yes | Yes | Yes | Yes | Yes | Yes | Yes | Yes | Yes | Yes | Yes |
| 6. Was the test/service/intervention clearly described and delivered consistently across the study population? | No | No | Yes | Yes | Yes | No | Yes | Yes | Yes | Yes | Yes | Yes |
| 7. Were the outcome measures prespecified, clearly defined, valid, reliable, and assessed consistently across all study participants? | Yes | Yes | Yes | Yes | Yes | Yes | Yes | Yes | Yes | Yes | Yes | Yes |
| 8. Were the people assessing the outcomes blinded to the participants' exposures/interventions? | No | No | No | No | No | No | No | No | No | No | No | No |
| 9. Was the loss to follow-up after baseline 20% or less? Were those lost to follow-up accounted for in the analysis? | Yes | Yes | Yes | Yes | Yes | Yes | Yes | Yes | Yes | Yes | Yes | Yes |
| 10. Did the statistical methods examine changes in outcome measures from before to after the intervention? Were statistical tests done that provided p values for the pre-to-post changes? | NA | NA | NA | NA | NA | No | NA | NA | NA | Yes | Yes | Yes |
| 11. Were outcome measures of interest taken multiple times before the intervention and multiple times after the intervention (i.e., did they use an interrupted time-series design)? | Yes | Yes | Yes | NA | Yes | NA | Yes | Yes | Yes | Yes | Yes | Yes |
| 12. If the intervention was conducted at a group level (e.g., a whole hospital, a community, etc.) did the statistical analysis take into account the use of individual-level data to determine effects at the group level? | NA | NA | NA | NA | NA | NA | NA | NA | NA | NA | NA | NA |

**Table S1. Quality Assessment tool for Before-After (Pre-Post) Studies With No Control Group of the studies included in the meta-analysis.** NA=Not Applicable.

1. Shea, J.J. and D.S. Postma, *Findings and Long-term Surgical Results in the Hearing Loss of Osteogenesis Imperfecta.* Archives of Otolaryngology, 1982. **108**(8): p. 467-470.

2. Pedersen, U. and O. Elbrønd, *Stapedectomy in osteogenesis imperfecta.* ORL, 1983. **45**(6): p. 330-337.

3. Garretsen, T.J.T.M. and C.W.R.J. Cremers, *Ear Surgery in Osteogenesis Imperfecta.* Arch Otolaryngol Head Neck Surg, 1990. **116**: p. 317-323.

4. Albahnasawy, L., A. Kishore, and B.F. O'Reilly, *Results of stapes surgery on patients with osteogenesis imperfecta.* Clinical Otolaryngology and Allied Sciences, 2001. **26**(6): p. 473-476.

5. Van der Rijt, A.J.M. and C.W.R.J. Cremers, *Stapes surgery in osteogenesis imperfecta: Results of a new series.* Otology and Neurotology, 2003. **24**(5): p. 717-722.

6. Kuurila, K., S. Pynnönen, and R. Grénman, *Stapes surgery in osteogenesis imperfecta in Finland.* Annals of Otology, Rhinology and Laryngology, 2004. **113**(3 I): p. 187-193.

7. Vincent, R., et al., *Stapedotomy in osteogenesis imperfecta: A Prospective Study of 23 consecutive cases.* Otology and Neurotology, 2005. **26**: p. 859-865.

8. Swinnen, F.K.R., et al., *Audiometric, surgical, and genetic finding in 15 ears of patients with osteogenesis imperfecta.* Laryngoscope, 2009. **119**(6): p. 1171-1179.

9. Swinnen, F.K.R., et al., *Stapes surgery in osteogenesis imperfecta: retrospective analysis of 34 operated ears.* Audiology & Neurotology, 2012. **17**(3): p. 198-206.

10. Vincent, R., et al., *Stapedotomy in osteogenesis imperfecta: A prospective study of 32 consecutive cases.* Otology and Neurotology, 2014. **35**(10): p. 1785-1789.

11. Skarżyński, H., et al., *Results of stapedotomy in otosurgical treatment of adult patients with osteogenesis imperfecta.* Auris Nasus Larynx, 2019. **46**(6): p. 853-858.

12. Ma, X., et al., *The impact of stapes surgery on osteogenesis imperfecta: a retrospective comparison of operative outcomes with those for patients with otosclerosis.* Acta Oto-Laryngologica, 2020: p. 1-9.
